# A new blue-tailed Monitor lizard (Reptilia, Squamata, *Varanus*) of the *Varanus indicus* group from Mussau Island, Papua New Guinea

**DOI:** 10.3897/zookeys.568.6872

**Published:** 2016-02-23

**Authors:** Valter Weijola, Stephen C. Donnellan, Christer Lindqvist

**Affiliations:** 1 Zoological Museum, University of Turku, 20014 Turku, Finland (VW); 2 South Australian Museum, North Terrace, Adelaide, 5000 and School of Biological Sciences, University of Adelaide, Adelaide 5005, Australia (SCD); 3 Cell Biology, Åbo Akademi University, 20520 Turku, Finland (CL)

**Keywords:** Melanesia, Bismarck Archipelago, St. Matthias islands, Varanidae, *Varanus doreanus*, *Varanus finschi*, *Varanus yuwonoi*, mitochondrial phylogeny, biogeography, taxonomy

## Abstract

We describe a new species of *Varanus* from Mussau Island, north-east of New Guinea. The new species is a member of the *Varanus indicus* species group and is distinguished from all other members by both morphological and molecular genetic characters. It is the third species of *Varanus* reported from the Bismarck Archipelago and the first record of a yellow tongued member of the *Varanus indicus* species group from a remote oceanic island. The herpetofauna of Mussau Island has not been well studied but the discovery of this new species is in accordance with recent findings indicating that the island may harbor several unknown endemic vertebrates. The distribution of the closely related *Varanus finschi* is also discussed in the light of recent fieldwork and a review of old records.

## Introduction

The varanid subgenus *Euprepiosaurus* Fitzinger comprises two species groups: the *Varanus indicus* and *Varanus prasinus* species groups. The subgenus is geographically restricted to a large region east of Wallace’s line with the Solomon Islands and parts of Micronesia forming the eastern and northern boundaries (Ziegler et al. 2007a, Sweet and Pianka 2007). The systematic arrangement is well-supported by molecular and morphological studies (Ziegler and Böhme 1997, Fitch et al. 2006, Vidal et al. 2012). Several new monitor lizards of the subgenus *Euprepiosaurus* have been discovered from islands in the southwest Pacific since the early 1990s. This increase has mainly been the result of taxonomic studies of museum collections, and the appearance of novel species through the international trade in live animals. Eleven species have been described from the Moluccas and Raja Ampat islands in eastern Indonesia, more often as a result of new specimens arriving through the animal trade rather than resulting from field studies and scientific collections (e.g. Böhme and Ziegler 1997, Harvey and Barker 1998).

Over the same time period, the monitors of Papua New Guinea and the Solomon Islands have received considerably less scientific attention. Papua New Guinea has no legal international live animal trade and it’s fauna is less represented in European museum collections. Since 1990 only two new species have been described from Papua New Guinea, both from revisions of colonial era museum collections: *Varanus telenesetes* (Sprackland 1991) (possibly a synonym of *Varanus bogerti* Mertens [Weijola obs.]), and *Varanus finschi* Böhme, Horn & Ziegler, 1994. As a consequence, the Melanesian islands have been considered less diverse in comparison to the Moluccas (Ziegler et al. 2007a).

As part of a larger survey of the monitors of the Bismarck Archipelago of Papua New Guinea in 2012, VW collected three specimens of a previously unknown blue-tailed species of the *Varanus indicus* species group from Mussau Island in the St Matthias group. Previously three individual monitor lizards in total had been recorded on two separate occasions from the St Matthias group - a juvenile specimen collected in 1944 (AMNH 85887) and two adult specimens collected during the Noona Dan Expedition in 1961-1962 (ZMUC 4272-4273) (identified as *Varanus finschi* in Philipp et al. [2007]).

Four species of the *Varanus indicus* species group (including the new taxon from Mussau) share the occurrence of yellow pigmentation on the tongue (Harvey and Barker 1998). Although taxon sampling in published molecular phylogenies has been limited, these yellow-tongued monitors have consistently formed a basal clade within the *Varanus indicus* species group (Ast 2001, Welton et al. 2013). *Varanus doreanus* Meyer is widespread on New Guinea, Aru, Biak, Waigeo, Salawati and parts of northern Cape York (Ziegler et al. 2007a). *Varanus finschi* Böhme, Horn & Ziegler, 1994 is likely endemic to New Britain (see discussion). *Varanus yuwonoi* Harvey & Barker is endemic to Halmahera and possibly nearby islands (Weijola 2010).

Molecular genetic and morphological studies of the newly collected material from Mussau Island clearly show the population represents a distinct taxon of yellow-tongued monitor. The concept best applicable to allopatric species is probably the Evolutionary Species Concept (ESC) (Simpson 1951) and more recent integrative approaches such as the Unified Species Concept (de Queiroz 2007). On account of its distinctive morphology, phylogenetic position and geographically isolated distribution we recognize the Mussau monitor as a unique evolutionary lineage and describe it as a new species herein.

## Materials and methods

**Taxonomy.** We follow the nomenclature of de Lisle (2009) for the taxa treated. The taxonomic identities of *Varanus cerambonensis* and *Varanus indicus* (*sensu* Philipp et al. 1999) included in the molecular phylogeny have recently been challenged (see Weijola and Sweet 2015, Weijola 2015) but until a ruling from the ICZN is issued we follow the nomenclature of Philipp et al. (1999).

**Morphology.** We obtained data for the meristic characters used by Brandenburg (1983) and in later works on the *Varanus indicus* group (e.g., Ziegler et al. 2007b, Weijola and Sweet 2010). Measurements were taken to the nearest 0.5 mm (head) or 1 mm with a steel tape or calipers. Comparative scale counts for *Varanus doreanus* and *Varanus yuwonoi* were taken from the literature (Brandenburg 1983, Harvey and Barker 1998, Ziegler et al. 2007b). Specimens listed in Brandenburg (1983) were identified by VW. We used PAST (Hammer et al. 2001) for Principal Components Analyses (PCA). The variance-covariance matrix was used on the unaltered scalation data including P, Q, S, T, X, XY, m, N and R characters. Definitions of, and abbreviations used for measurements, proportion indices and scale counts are presented in Table 1.

**Table 1.** Definitions of, and abbreviations used for measurements, proportion indices and scale counts.

Symbol	Description
Measurements	
SVL	Snout to vent length
F	tail length
TL	total length
E	body length from gular fold to cloaca
D	head-neck length from tip of snout to gular fold
A	head length from snout to anterior dorsal margin of tympanum
B	head width at maximum span of postorbital arch
C	head depth at midpoint of orbit
G	facial length from center of nostril to anterior margin of orbit
H	snout length from tip of snout to center of nostril
I	temporal length from anterior margin of eye to anterior border of tympanic recess
Proportion Indices	
1	relative tail to body length - F/SVL
2	relative position of nostril to eye - G/H
9	relative position of nostril to tip of snout - [A-I]/G
10	relative head length to width - A/B
11	relative head length to height - A/C
Scale Counts	
S	Midbody scale rows
XY	dorsal scale rows from dorsal margin of tympanic recess to anterior margin of hind limbs
T	transverse rows of mid-ventral scales from gular fold to anterior margin of hind limbs
X	transverse rows of dorsal scales from posterior margin of tympanic recess to gular fold
m	scales around neck at anterior margin of gular fold
N	rows of mid-ventral scales from tip of snout to gular fold
P	scales from rictus to rictus across dorsum of head
Q	scales around tail base
R	scales around tail counted at 1/3 of the length from the base
DOR	number of dorsal scalerows from the last occipital scale to a point dorsal to the posterior margin of the cloaca
VEN	Number of mid-ventral scales from the gular fold to the anterior margin of the cloaca

Museum abbreviations used are: ABTC: Australian Biological Tissue Collection (South Australian Museum, Adelaide), AMNH: American Museum of Natural History (New York), AMS: Australian Museum (Sydney), BPBM: Bernice Pauahi Bishop Museum (Honolulu), NMW: Naturhistorische Museum Wien (Wien), QM: Queensland Museum, RMNH: Naturalis museum (Leiden), UMMZ: Museum of Zoology, University of Michigan, ZMA: Zoological Museum of the University of Amsterdam (currently Naturalis), ZMB: Zoologische Museum der Humboldt Universität (Berlin), ZMUC: Zoological Museum, University of Copenhagen, and ZMUT: Zoological Museum, University of Turku.

**Molecular genetic methods.** A 661 bp fragment of the mitochondrial genome, including the 3’ end of the NADH dehydrogenase subunit 4 (*ND4*) gene (710 bp) and the 5’ end of *tRNA^His^* (64 bp) gene, was amplified and sequenced (hereafter referred as *ND4*) using the forward primer 5’- TGA CTA CCA AAA GCT CAT GTA GAA GC-3’ (Forstner et al. 1995) with the reverse primer 5’ CAT TAC TTT TTA CTT GGA TTT GCA CCA-3’ (Arévalo et al. 1994). A 566 bp fragment of the mitochondrial *16S rRNA* gene was amplified and sequenced using the forward primer: 5’ - CGC CTG TTT ATC AAA AAC AT - 3’ with the reverse primer: 5’ - CCG GTC TGA ACT CAG ATC ACG T – 3’ (Palumbi et al. 1991).

The amplification reactions were performed in a final volume of 50ul using the Phusion U Hot Start PCR Master Mix (ThermoFisher Scientific, St. Leon-Rot, Germany). The PCR profile for the *ND4* amplification was 9 min at 94 °C (initialization step, one cycle), 30 sec at 94 °C (denaturation step, 35 cycles), 25 sec at 46,5 °C (annealing step, 35 cycles), 35 sec at 72 °C (extension step, 35 cycles) and 2 min at 72 °C (final elongation step, 1 cycle). The corresponding profile for the *16S rRNA* amplification was 9 min at 94 °C (initialization step, one cycle), 30 sec at 94 °C (denaturation step, 35 cycles), 25 sec at 55 °C (annealing step, 35 cycles), 35 sec at 72 °C (extension step, 35 cycles) and 2 min at 72 °C (final elongation step, 1 cycle). A negative control (no template present) was also included in all PCRs. All PCR products were analyzed by gel electrophoresis on a 1.8% agarose gel containing 0.5 µg/ml ethidium bromide (Promega, Madison, USA) before they were sequenced.

PCR products were sequenced by the Beckman Coulter Genomics company (Essex, UK). GenBank accession numbers of the new sequences are provided in Table 2.

**Table 2.** Specimens examined morphologically (*), or sequenced for mtDNA. Voucher registration numbers (#), collection localities and GenBank accession numbers are listed.

Species	Voucher Registration #	Collection Locality	GenBank *ND4, 16S RNA*
*Varanus cerambonensis*	WAM R109448	Banda Is., Ind.	KU513445, KU513465-
*Varanus cerambonensis*	WAM R109476	Banda Is., Ind.	KU513446, KU513466
Varanus doreanus*	AMS R28680	Gamog, Karkar Is. PNG	-
Varanus doreanus*	AMS R25686	Gamog, Karkar Is. PNG	-
Varanus doreanus*	AMS R25687	Gamog, Karkar Is. PNG	-
Varanus doreanus*	AMS R129210	Jama, East Sepik Prov., PNG	-
Varanus doreanus*	BPBM 19509	Mt Obree, Northern Prov., PNG	KU513447, KU513467
Varanus doreanus*	Naturalis ZMA10190	? Indonesia	-
Varanus doreanus*	Naturalis ZMA10193	Sabang, West Papua, Ind.	-
Varanus doreanus*	Naturalis ZMA10194a	Noord River, West Papua, Ind.	-
Varanus doreanus*	Naturalis ZMA10195	Wendessi, West Papua, Ind.	-
Varanus doreanus*	Naturalis ZMA10199	Sermonai River, West Papua, Ind.	-
Varanus doreanus*	Naturalis ZMA12125	Hollandia (Jayapura), Papua, Ind.	-
Varanus doreanus*	Naturalis RMNH5164	Digoel River, West Papua, Ind.	-
Varanus doreanus*	Naturalis RMNH7035	Manokwari	-
Varanus doreanus*	Naturalis RMNH21029	Gariau-lake jamoer, West Papua, Ind.	-
Varanus doreanus*	Naturalis RMNH21051	Fak Fak, West Papua, Ind.	-
Varanus doreanus*	Naturalis RMNH21055b	Manokwari, West Papua, Ind.	-
Varanus doreanus*	QM J15363	Cape York, Qld. Aus.	-
Varanus doreanus*	QM J18103	Claudie River, Qld, Aus.	-
Varanus doreanus*	QM J32020	Pascoe River, Qld. Aus.	-
*Varanus doreanus*	UMMZ 227117	Merauke, Papua, Ind.	KU513448, KU513468
Varanus finschi*	AMS R5618	Duke of York, East New Britain, PNG	-
Varanus finschi*	AMS R129614	Amelei, New Britain, PNG	-
Varanus finschi*	ZMUT Sa186	Nodup, New Britain, PNG	KU513443, KU513463
Varanus finschi*	ZMUT Sa190	near Kokopo, New Britain, PNG	KU513444, KU513464
*Varanus finschi*	MNHN 00 192	Blanche Bay, New Britain, PNG	-
*Varanus finschi*	MNHN 00 195	Blanche Bay. New Britain, PNG	-
*Varanus indicus*	ZMUT Sa191	Normanby Is., PNG	KU513455, KU513476
*Varanus indicus*	ZMUT Sa202	New Britain, PNG	KU513456, KU513477
*Varanus indicus*	No voucher, tissue QM A002919	Peach Creek, Qld, Aus.	KU513452, KU513473
*Varanus indicus*	WAM R109525	Aru Islands, Ind.	KU513453, KU513474
*Varanus indicus*	WAM R109551	Aru Islands, Ind.	KU513454, KU513475
*Varanus indicus*	No voucher, tissue ABTC13465	Maningrida, NT, Aus.	DQ525167, KU513469,
*Varanus indicus*	AMS R137997	Fergusson Is., PNG	KU513450, KU513471
*Varanus indicus*	LSUMZ H10449	Wewak, East Sepik Prov., PNG	KU513451, KU513472
*Varanus jobiensis*	AMS R115341	Doido, Chmbu Prov.,PNG	DQ525163, KU513478
*Varanus jobiensis*	AMS R116999	Wigote, Sandaun Prov., PNG	KU513457, KU513479
*Varanus melinus*	UMMZ 222682	Sula Islands, Ind.	KU513458, KU513480
*Varanus prasinus*	AMS R115500, ZFMK 70600	Mt Boobiari, Sandaun Prov., PNG. West Papua, Ind.	DQ525171, EF193687
Varanus semotus*	ZMUT Sa176	Mussau Is., PNG	KU513459, KU513482
Varanus semotus*	ZMUT Sa177	Mussau Is., PNG	KU513460, KU513483
Varanus semotus*	ZMUT Sa178	Mussau Is., PNG	KU513461, KU513484
Varanus semotus*	ZMUC 4272	Talumalaus, Mussau Is., PNG	-
Varanus semotus*	ZMUC 4273	Talumalaus, Mussau Is., PNG	-
*Varanus yuwonoi*	UMMZ 225545	Halmahera, Ind.	KU513462, KU513481

**Phylogenetic analysis.** Resulting sequences were aligned by MUSCLE (Edgar 2004) as implemented in GENEIOUS v8.1.4 and concatenated for phylogenetic analysis. Bayes factors were used to assess all possible alternative partitioning strategies for five data subsets: 1st, 2nd and 3rd codon positions, the tRNA and *16S rRNA* in PartitionFinder v1.0.0 (Lanfear et al. 2012). The Akaike Information Criterion (AIC) and Bayes Information Criterion (BIC) were used to assess the best fit partition strategy and nucleotide substitution model for each data subset in the selected partition strategy. Sequences were analysed phylogenetically using Bayesian and maximum likelihood (ML) methods. Bayesian analysis was conducted using MrBayes v3.2.5 (Ronquist and Huelsenbeck 2003). The analysis was run with model parameters unlinked using default priors for ten million generations with two independent runs and two chains sampling every 500 generations. The first 25% of sampled trees were discarded as burn-in and convergence was assessed by examining effective sample sizes (ESS values), split frequencies of clades across runs and likelihood plots through time in TRACER v1.6 (Rambaut and Drummond 2007). Evolutionary trees were constructed with the ML criterion of optimality implemented in the web server version of RAxML (Stamatakis et al. 2008), which uses the GTR+Γ model of nucleotide substitution. The robustness of phylogenetic hypotheses was tested with non-parametric bootstrapping. *Varanus prasinus*, from the sister lineage to the *Varanus indicus* species group, was used as outgroup.

Net average sequence divergence between lineages (*dA*) was calculated from the *ND4* data only in MEGA v5 (Tamura et al. 2011) as: *d*A = *d*XY – (*d*X + *d*Y)/2, where, *d*XY is the average distance between groups X and Y, and *d*X and *d*Y are the within-group means. Net average sequence divergence was calculated more broadly for sister species pairs of *Varanus* where more than one sequence was available for each member of the pair from our data and the data of Fitch et al. (2006), Smith et al. (2007), Smissen et al. (2013), Maryan et al. (2014), Doughty et al. (2014) and GenBank accessions for *Varanus komodoensis*.

## Results

### Varanus semotus

Taxon classificationAnimaliaSquamataVaranidae

Weijola, Donnellan & Lindqvist sp. n.

http://zoobank.org/B5D753CF-7C2F-42B4-A7FE-376F0E8FCF6A

Figs 1 , 2 , 3

#### Holotype.

**Figure 1.**
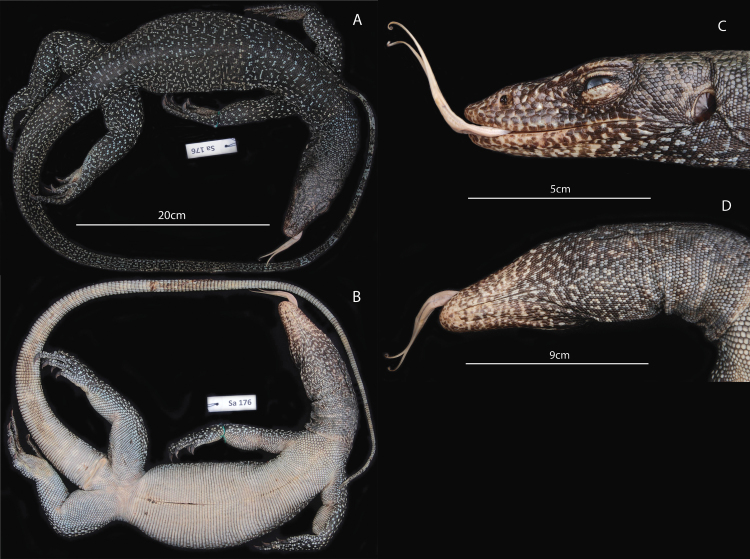
**A–D** Full body dorsal, ventral, head profile and gular region of the holotype of *Varanus semotus* -ZMUT Sa176.

**Figure 2.**
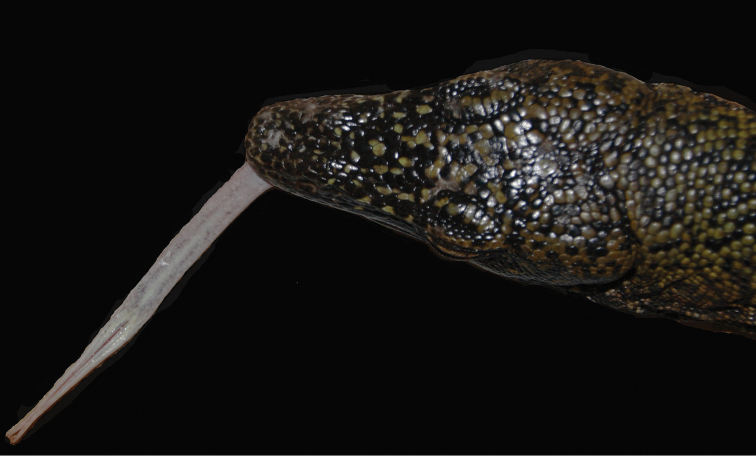
Tongue color of the freshly collected *Varanus semotus* holotype ZMUT Sa176.

ZMUT Sa176 (field nr. 60) (Figs 1–2) collected by Valter Weijola just north of the village of Nai, 30 September 2012, 2m elev. Mussau Island, St. Matthias group, Papua New Guinea, latitude -1.525, longitude. 149.749.

#### Paratypes.

ZMUT Sa177 (field nr 64), ZMUT Sa178 (field nr 66) collected by Weijola near Nai 4 and 7 October 2012. Mussau Island, Papua New Guinea, latitude -1.525, longitude 149.749, ZMUC 4272 (field number E192) and ZMUC 4273 (field number E282) collected by the Noona Dan Expedition (presumably by Søren Andersen) on 19 January and 5 February 1962 at Talumalau, Mussau Island, Papua New Guinea.

#### Other material.

AMNH 85887 collected by John Gardiner in 1944, St Matthias Islands, Papua New Guinea.

#### Etymology.

The specific epithet *semotus* is Latin for distant or remote and refers to the isolated occurrence on Mussau, separated by several hundred kilometers from its closest relatives. The term is employed as a masculine adjective.

#### Diagnosis.

**Figure 3.**
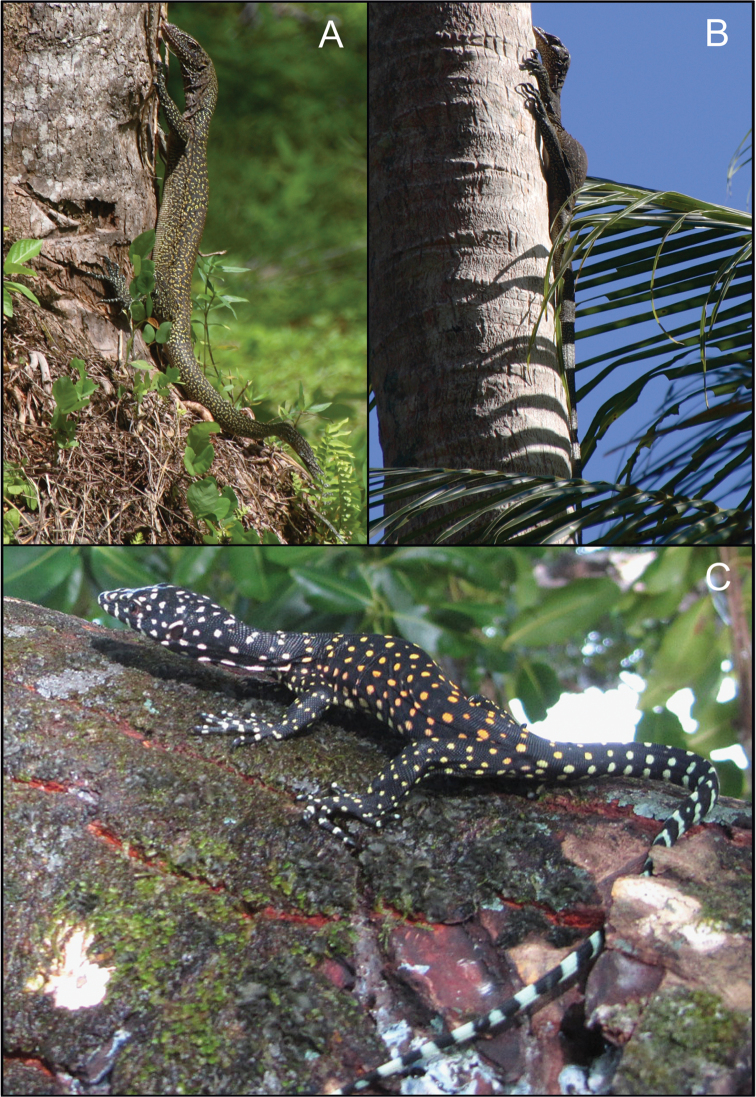
**A–C** Images of live *Varanus semotus* at Nai on Mussau Island. **A** an adult in its habitat at the outskirts of Nai **B** an adult basking on the trunk of a palm tree (photos by VW), and **C** a juvenile (photo by Quetzal Dwyer).

*Varanus semotus* sp. n. is distinguished from all other species of *Varanus* by a combination of the following characters. (1) Tongue white/pinkish to pale yellow (white in preservative) occasionally with small patches of dark pigmentation, the yellow pigment concentrated along the mid-dorsal line and the dorsal surface of the tines (Fig. 2). (2) Gular region marbled in black and cream-white. (3) The tail of adult individuals is indistinctly banded on the distal half, with a varying degree of turquoise to bluish pigmentation on the distal 2/3. (4) Juveniles are black with white spots on the head, yellow and orange spots on the dorsum, and have well defined cream colored to pale greenish tail bands (Fig. 3C). (5) The number of dorsal scales, XY, ranges from 149 to 153. (6) The number of midbody scale rows, S, ranges from 152 to 161. (7) The dorsum is black with single- and clustered groups of dispersed yellow/orange scales. (8) There are several complete rows of paryphasmata across the asulcal side of the hemipenis below the lobes. (9) Geographical distribution restricted to Mussau Island.

#### Comparisons.

**Figure 4.**
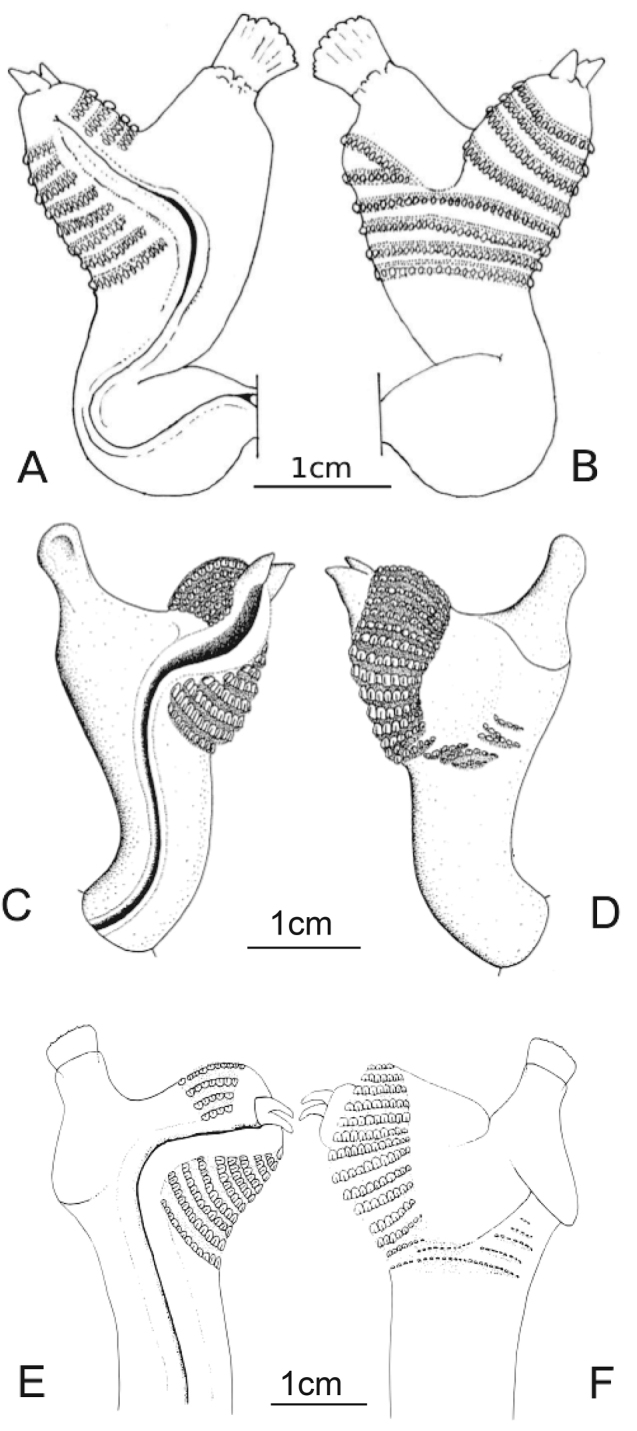
Drawings of the hemipenises of the male paratype ZMUT Sa178 of *Varanus semotus*, sulcal (**A**) and asulcal (**B**) (illustration by Sam Sweet), *Varanus doreanus* (ZFMK 26341) sulcal (**C**) and asulcal (**D**), and *Varanus finschi* (ZMB 14596) sulcal (**E**) and asulcal view (**F**) (**C–F** illustrated by Thomas Ziegler, reproduced from Ziegler et al. 1999).

*Varanus semotus* sp. n. is a member of the *Varanus indicus* species group of the subgenus *Euprepiosaurus* distinguished by the asymmetrical sulcus spermaticus and laterally compressed tail (Ziegler et al. 2007a). Within the *Varanus indicus* species group it can be distinguished from all species except for *Varanus doreanus*, *Varanus finschi* and *Varanus yuwonoi* by the presence of yellow pigmentation on the tongue. *Varanus semotus* is unlikely to be confused with any other species except for *Varanus doreanus*, from which it can be difficult to distinguish by external morphology. On average, *Varanus semotus* has lower XY (149–153 vs. 153–215) and S (152–161 vs. 158–180) scale counts than *Varanus doreanus. Varanus semotus* exhibit several complete rows of paryphasmata crossing the asulcal side of the hemipenis while this is restricted to the medial part of the trunk and lobes on *Varanus doreanus* (Fig. 4). In contrast to the morphological similarity of these two species, they show a significant genetic separation: 11.5% mean net sequence divergence (*dA*) (Table 5B). *Varanus semotus* is readily distinguished from *Varanus finschi* and *Varanus yuwonoi*, both of which have predominately white to cream colored throats and considerably higher scalecounts (S over 170, XY over 165). Additionally, *Varanus finschi* lacks blue pigmentation on the tailand exhibits transverse rows of yellow ocelli on the dorsum. Furthermore, *Varanus finschi* and *Varanus semotus* have a *dA* of 6.4% (Table 5A). *Varanus yuwonoi* has a unique color pattern being predominantly black on the anterior 1/3 of the body, yellow on the lower back and tailbase, and with a blue tail. Furthermore, *Varanus yuwonoi* and *Varanus semotus* have a *dA* of 11.6% (Table 5B).

#### Description of the holotype.

A female of a total length of 1010mm (SVL: 390mm, F: 620mm). The specimen is well preserved and has an incision running from below the rib-cage to the lower abdomen. There are unhealed lacerations on the ventral part of the tail at around midlength, possibly from a dog bite. The ground color of the dorsal aspect of the body, tail, head and limbs is black. The tail is long and slender, 1.59 times as long as the body, and 38.75 times as long as it is high (16mm) at midlength. It is rounded at the base, becoming increasingly laterally compressed distally starting at 60mm from the base. Two to five middorsal caudal scale rows form a double ridge extending from 1/8 of its length and distally almost to the tip. There are nine discernible blue crossbands each about 6–9 scale rows wide on the distal half of the tail with intermediate blue markings. The ventral scales are white to cream colored with a narrow line of dark brown pigmentation running along the anterior margin. The gular region is dark brown-black and marbled with yellowish and greyish scales. The nostrils are large and round, positioned closer to the snout than the anterior margin of the eye. Nasal capsules expanded forming a groove on the rostrum. The tongue is whitish (in preservative) with small spots of grey-blue pigmentation along the lateral margins. The teeth are long, sharp and only slightly recurved. The limbs are muscular, claws dark-brown and recurved. The head is dark-brown to black and covered with irregular brown-grey markings.

Nuchal scales are slightly domed to flattened, elongate to polygonal immediately behind the head becoming round to oval towards the shoulders and with 1–10 scale pits. Gular scales flattened, round to irregularly polygonal, equipped with 1–5 pits and sometimes bordered by incomplete rows of granules. Mental scales irregular in shape from rectangular to polygonal and elongate. Dorsal scales slightly elongated, rounded or polygonal and with a low central keel. Most are surrounded by an incomplete row of granular scales and with one or two pits located near the posterior end.

Laterodorsal scales are smaller, round, slightly domed, surrounded by granules and with one to three pits. Middorsal caudal scales rectangular, elongate, with a single pit at the posterior end, and lack granules. Mid-ventral caudals twice as long as mid-dorsal caudal scales, elongate and keeled.

Suprafemorals and suprabrachials oval, keeled and surrounded by 1–2 rows of granules. Supratibials irregularly round to oval, polished or keeled and surrounded by 2–3 rows of granules. Infrafemorals round to slightly oval and usually equipped with a row of granules along the distal edge. Infratarsals round to polygonal, highly domed and with a few granules around the corners. Most are light in color and only few have dark pigmented centers. There are rows of 9 enlarged postdigital scales along the outer margin of the fourth hind toe. Infracarpals similar in color to infratarsals, round to slightly polygonal, domed and with granules around the corners.

Dorsal head scales irregularly sized and polygonal, flattened, and equipped with numerous pits. There are seven enlarged supraocular scales on each side, bordered by 1-3 rows of smaller scales. Rostral scale, paired. There are 25+25 enlarged pentagonal supralabial scales equipped with as much as 30 pits. There are 26+26 irregularly shaped infralabials densely covered with pits. Temporal scales square or polygonal, polished and covered with up to ten pits. Two rows of scales separate the supralabials from the nostrils. The occipital scale is enlarged and roundish.The scales on the chest are enlarged, irregularly polygonal, flat and surrounded by only few granules. Ventral scales from the lower chest and down to the abdomen are rectangular, irregularly elongate, bordered by granules along the posterior margin, and with a single pit at the posterior end. The oviducts are translucent white and contains series of ovarian follicles about 10–15mm long.

#### Scale counts, measurements and proportion indices of the type series.

Are presented in Table 3.

**Table 3.** Measurements, proportion indices and scalecounts of the type series of *Varanus semotus*.

Measurements	ZMUT Sa176 (holotype)	ZMUT Sa177 (paratype)	ZMUT Sa178 (paratype)	ZMUC 4272 (paratype)	ZMUC 4273 (paratype)
SVL	390	400	400	45	48
F	620	610	640	69	69
TL	101	101	104	114	117
E	236	228	235	-	-
D	135	140	150	-	-
A	66	68.5	70	78	80
B	39	39	40.5	48	48
C	27	24	26.5	32	34
G	19	21	23	25	26
H	14	14	14	16	17
I	33	33.5	35	-	-
**Proportion indices**					
1	1.59	1.53	1.6	1.53	1.44
2	1.36	1.5	1.64	1.56	1.53
9	1.74	1.67	1.52	-	-
10	1.69	1.76	1.73	1.63	1.67
11	2.44	2.85	2.64	2.44	2.35
**Scalation**					
S	161	162	152	167	160
XY	153	147	149	150	152
T	89	87	87	89	89
X	40	39	38	39	43
N	93	89	85	92	91
m	116	114	108	119	118
P	47	47	47	49	51
Q	100	97	99	103	103
DOR	166	162	164	165	164
VEN	107	108	105	110	113

#### Hemipenal morphology.

The hemipenis of the male paratype ZMUT Sa178 was everted prior to fixation (Fig. 4). The trunks are dark grey pigmented on the asulcal side excluding the lobes. The sulcus spermaticus runs medially on the trunk, turns to the lateral lobe and deflates at the base of the hemibaculum. There are four paryphasmata rows running across the asulcal side of the trunk proximally to the bifurcation of the lobes. About seven additional rows of paryphasmata continues up on the lateral lobe towards the apex. Two rows of paryphasmata runs on the lateral side of the medial lobe as a continuation of the truncal ornamentation. The medial hemibaculum is ossified, quadrangular and slightly decurved. The lateral hemibaculum is smaller, triangular, and with two sharp ends.

#### Variation and color in life.

The type series is relatively uniform in coloration and pattern. The ground color of the dorsum, tail, legs and head is black. The dorsum and femurs are densely covered by yellow-orange scales, most aggregated in groups of 1–10 (mostly 2–4) scales forming lines, half circles or more rarely complete rings. The markings becomes denser on the neck and changes in color to brown-grey-yellow on the upper neck and head. On the dorsal side of the hands, feet, digits, supratibials and distal 2/3 of the tail most of the light markings are of a blue-green color. On the distal half of the tail these are arrayed in several indistinct transversal bands. The venter is white-pinkish, and with a blue hue on the infratibial surfaces. The upper chest and gular region has an orange-pink hue and is densely marmorated with black on the anterior half. The black markings are paler half adjacent to the gular fold. Photographs from the field allows for a description of coloration of a juvenile (Fig. 3C). This specimen is black with bright orange and yellow spots on the dorsum, white spots from the shoulder and anteriorly, more or less arrayed in 16 transverse rows between the venter and the head. On the distal 2/3 of the tail these spots turns into 16 complete, well defined whitish crossbands. On the dorsal sides of the legs and around the tailbase the spots are yellow-green. The head is decorated with white patches, and the lips have five white bars on both sides. The iris is dark brown.

#### Distribution.

**Figure 5.**
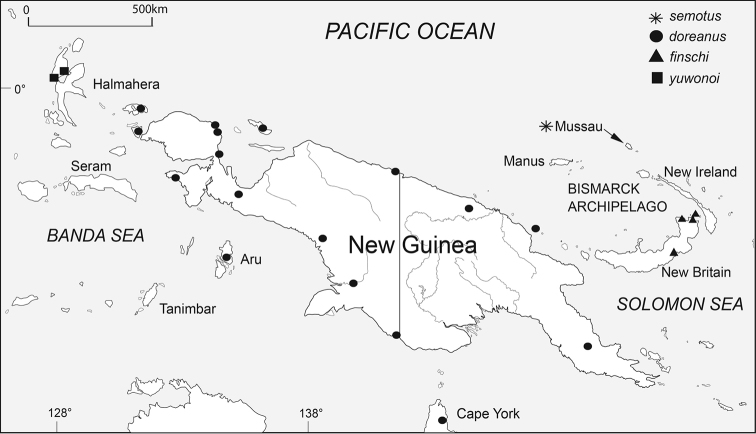
Map of New Guinea and surrounding islands showing the distribution of the members of the yellow-tongued monitors.

*Varanus semotus* is known so far only from Mussau, an island of 414 km^2^ in the northern Bismarck Sea (Fig. 5). According to some of the locals on Mussau, monitors are absent from Emirau, the second largest island of the St. Matthias group, but this needs confirmation from fieldwork. It is also unknown whether this species occurs on the other two nearby islands Emananus and Eloaua.

#### Natural history.

**Figure 6.**
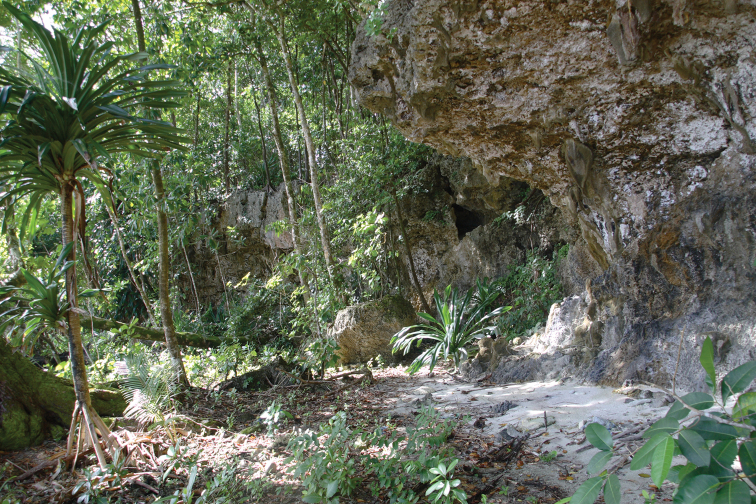
Typical vegetation of coastal karst areas of Mussau Island where several *Varanus semotus* were observed (photo by VW).

A total of 16 observations were made during fieldwork on Mussau, all of them along the coast near the village of Nai at the SE corner of the island. Searches in the secondary growth forest of the interior of the island and in the mangrove forests near Palakau did not produce any observations. The relatively dry coastal vegetation near Nai comprises a mixture of coconut palms, pandanus and other trees and shrubs able to persist in the karst, limestone and salt spray affected area (Fig. 6). In this vegetation type monitors appeared to be relatively common. Just south of the village there is a freshwater spring with a small area of Sago palms which was also a popular site for monitors. The lizards were usually spotted either as they were foraging on the ground and quickly fled up in trees, or while they were basking on the trunks of palms or other trees. The specimens collected as vouchers were noosed from trees with a long pole. As is typical of the closely related *Varanus doreanus*, *Varanus finschi* and *Varanus yuwonoi* the specimens were exceedingly aggressive and inclined to bite when captured and handled. Stomach content analysis of the three ZMUT specimens revealed a total of five reptile eggs (3,2,0) and one small skink. All stomachs contained the remains of crabs. Philipp et al. (2007) recorded a bird as the stomach content of ZMUC 4272.

#### Morphology.

**Figure 7.**
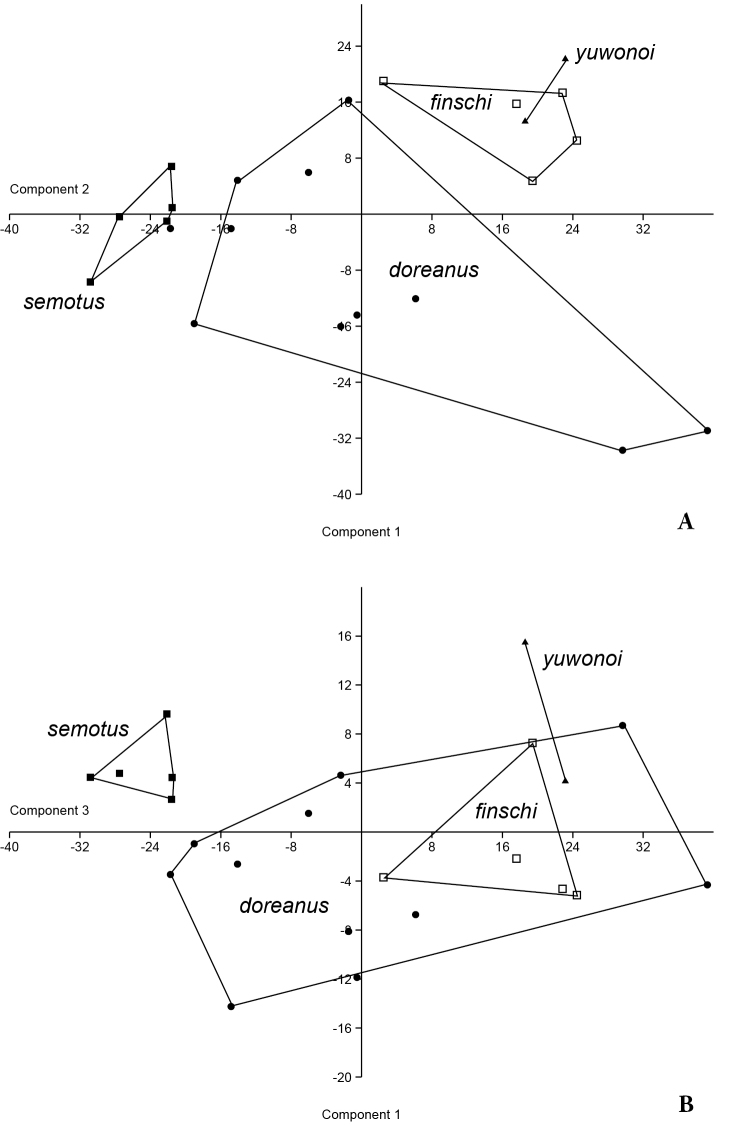
Principal Components Analysis of 9 scalation characters of the yellow-tongued monitors showing axis 1–2 (**A**) and 1–3 (**B**). Voucher information and scale counts are found in Appendix.

The PCA resolved group structure and only partly overlapping morpho-areas for the four species included (Fig. 7ab). *Varanus semotus* shows no area overlap on component axes 1–2 and 1–3 while the other three species show full or partial overlap on axes 1–3 (Fig. 7b). Potential sexual dimorphism in scalation characters have not been reported and were not taken into account. PC1 and PC2 accounted for over 80% of the variation with highest loadings on characters S, XY and m (Table 4). *Varanus yuwonoi* and *Varanus finschi* associate closely as a result of the mutually high scale counts. The population from Mussau is at the opposite extreme with lower scale counts than the other members. *Varanus doreanus*, for which the largest sample size was available (all from West Papua), demonstrate a considerable amount of intraspecific variation.

**Table 4.** Factor loadings, proportion of variance and eigenvalues for the three first components in the PCA. The two highest loading factors on each component are shown in bold.

Factor	Comp 1	Comp 2	Comp 3
P	0.019	-0.017	-0.312
Q	0.023	0.279	**0.548**
S	**0.291**	0.427	-0.304
T	0.141	0.292	0.292
XY	**0.861**	-0.170	0.083
m	0.198	**0.653**	0.362
N	0.143	0.222	0.222
R	-0.136	**0.457**	**0.457**
Proportion of variance	54%.2	29.1%	6.4%
Eigenvalue	435.4	233.9	51.6

#### Molecular genetic analysis.

**Figure 8.**
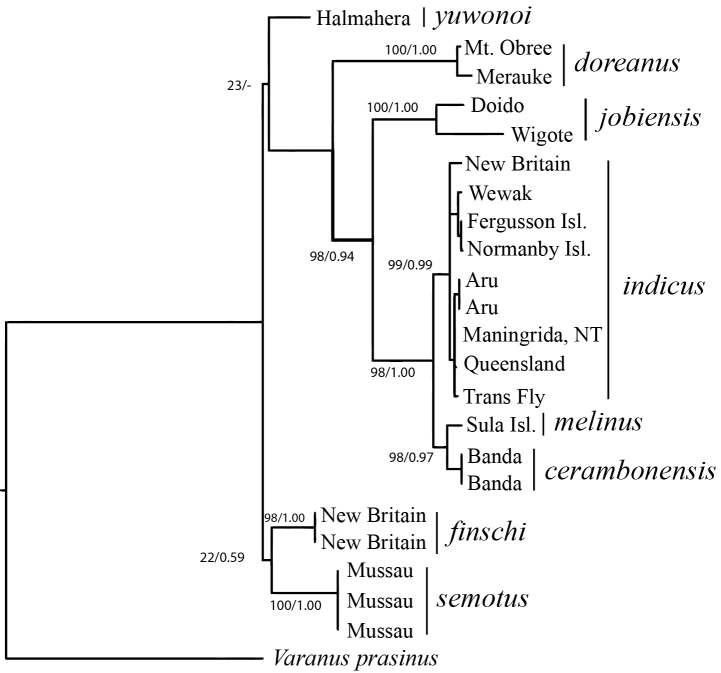
RaxML phylogeny of the Pacific monitors based on the combined mitochondrial *16S rRNA* and *ND4* regions; values show maximum likelihood bootstrap- and Bayesian posterior node support. Voucher information and GenBank accession numbers are presented in Table 2.

Using PartitionFinder, we selected three data partitions: *16S rRNA* + *ND4* 1^st^ codon positions + *tRNA^HIS^*, *ND4* 2nd codon positions and *ND4* 3rd codon positions with the following nucleotide substitution models respectively: TrN+G, HKY+I and TrN. Bootstrap proportions and Bayesian posterior probabilities strongly supported monophyly of conspecific sequences for each taxon where we had more than one sequence available (Fig. 8). Relationships between the taxa were also strongly supported for the most part except for the nodes placing *Varanus finschi*, *Varanus semotus* and *Varanus yuwonoi*, which effectively comprise a polytomy along with a clade comprising the remaining taxa.

A single haplotype was observed for the concatenated *16S rRNA* and *ND4* sequences among the three *Varanus semotus* sequenced. Net average uncorrected sequence divergence (*dA*) between *Varanus* sister species pairs for *ND4* ranged from 1.9% to 14.3% with a mean of 8.7% (Table 5). Net average uncorrected sequence divergence was 6.4% between *Varanus finschi* and *Varanus semotus* sp. n. and 2.3% between *Varanus cerambonensis* and *Varanus melinus*.

**Table 5.** Net average sequence divergence (*dA*) A) between sister species pairs of varanids and B) among members of the *Varanus indicus* species group.

**A**	
**Sister species pair**	***dA (%)***
*Varanus finschi-semotus* sp. n.	6.4
*Varanus cerambonensis-melinus*	2.3
*Varanus brevicauda-sparnos*	13.4
*Varanus eremius-sparnos*	14.3
*Varanus brevicauda-eremius*	8.5
*Varanus komodoensis-varius*	12.5
*Varanus mitchelli-semiremex*	12.1
*Varanus gouldii-rosenbergi*	11.2
*Varanus bushi-gilleni*	6.6
*Varanus pilbarensis-hamersleyensis*	6.3
*Varanus acanthurus insulanicus-baritji*	1.9

**B**								
**Taxon**	**c**	**i**	**d**	**f**	**m**	**s**	**y**	**j**
*Varanus cerambonensis* (c)	-							
*Varanus indicus* (i)	3.4	-						
*Varanus doreanus* (d)	11.5	11.4	-					
*Varanus finschi* (f)	11.7	11.1	11,4	-				
*Varanus melinus* (m)	2.3	3.6	12.3	12.9	-			
*Varanus semotus* (s)	10.2	10.2	11.5	6.4	11.1	-		
*Varanus yuwoni* (y)	11.1	11.7	11.7	7.0	12.6	6.7	-	
*Varanus jobiensis* (j)	6.8	6.2	9.4	8.6	7.4	8.3	8.9	-

## Discussion

**Biogeography.** The members of the *Varanus indicus* species group have been extraordinarily successful at colonizing the islands of the SW Pacific. *Varanus indicus* and its closest relatives, which are adept at oversea dispersal, have reached most islands between the western Moluccas and eastern Solomon islands. The yellow-tongued monitors on the other hand have, been far less adept at oversea dispersal. *Varanus doreanus* populations are with few exceptions (such as Biak) restricted to the land bridge islands of New Guinea. *Varanus yuwonoi* to Halmahera, a geologically complex island which was much more closely associated with parts of western New Guinea during the Miocene and Pliocene (Hall 1998) when it may have been easier to colonize by monitors and other terrestrial animals. *Varanus finschi* likely reached the nearby New Britain through oversea dispersal as this island has no known historical landbridges to New Guinea. *Varanus semotus* is notable as it is separated from its closest relatives by hundreds of kilometers of open sea and must have colonized the oceanic Mussau Island through long distance oversea dispersal, most likely by rafting. Vidal et al. (2012) estimate the age of *Varanus indicus* species group at around 6–11.5 mya. With this time reference the subsequent lineage diversification of species group should have occurred sometime in the late Miocene to early Pleistocene during which it is also likely that Mussau was colonized.

The St. Matthias group is situated on northern arc of the Bismarck Archipelago and has never had land connections to larger landmasses. It has three known endemic species of passerine birds; the Mussau monarch (*Symposiachrus menckei*), the Mussau triller (*Lalage conjuncta*) and the Mussau fantail (*Rhipidura matthiae*), but this number was most likely greater prior to human colonization (Steadman and Kirch 1998). There are no known native terrestrial mammals on Mussau but three still undescribed species of bats have recently been discovered (Flannery 1995, Aplin et al. 2015). Very little has been published on the herpetofauna of Mussau (e.g. Brown 1955, Mys 1988, Richards and Aplin 2015) and most of the recorded species are either widespread tramp species or endemics shared by Mussau and Manus. A recent (2014) faunal survey conducted by the Wildlife Conservation Society discovered a new endemic species of frog of the genus *Cornufer* (which constitute half of the known amphibian fauna). All nine species of skinks (single species of *Carlia*, *Eugongylus*, *Lamprolepis*, *Lipinia* and *Sphenomorphus* and 4 species of *Emoia*) recorded by the same expedition are widespread while one of the four species (2 *Gehyra*, 1 *Gekko* and 1 *Nactus*) of gekko (*Gehyra* sp.) is reported to be a new species endemic to Manus and Mussau Island (Richards and Aplin 2015). According to Richards and Aplin (2015) it is likely that additional species occur in the still unexplored fragments of primary forest of the interior. For now *Varanus semotus* is the only endemic lizard known from Mussau.

The absence of *Varanus indicus* s.l. which is otherwise almost universal on islands in the Southwest Pacific, including Manus and New Hanover, is more difficult to explain. The lack of widespread mangrove swamps around the coastlines seems an insufficient explanation as most island populations of Mangrove monitors are habitat generalists that occur in various coastal and inland habitat types (Weijola and Sweet 2015).

***Varanus finschi*.** Virtually nothing has been published on the biology of *Varanus finschi* since its initial description over two decades ago. In 1988 SCD collected a specimen at Amelei on the south coast of New Britain (AMS 129614). In 2012 VW visually identified four and collected two specimens in the vicinity of Rabaul, Kokopo and Nodup at the northern end of East New Britain (ZMUT Sa186 & 190). These new samples allowed us to include the species in a larger molecular phylogeny of the *Varanus indicus* group for the first time. The samples of alleged *Varanus finschi* (BPBM 17250 & 19510) from Milne Bay Province used by Ziegler et al. (2007) were re-identified as *Varanus cf. jobiensis* (by VW). Examination of live specimens also showed that the tongue color of *Varanus finschi* is yellow rather than pink/light as reported earlier (Sprackland 1997, Harvey and Barker 1998). According to VW’s field observations *Varanus finschi* is most numerous along the coast. Attempts to find monitors higher up in the Baining Mountains (500-700 m. elev.) were unsuccessful despite local testimonies of occasional observations. *Varanus indicus* is common along the coast and in the mangroves of New Britain and there appears to be at least partial habitat overlap between the two species.

*Varanus finschi* has been reported to have an extensive range outside of New Britain including New Ireland, New Guinea (Ziegler et al. 1999), northern Australia (Ziegler et al. 2001) and the Kei Islands (Philipp et al. 2004). However, as the only records from New Guinea (ZMB 18838 & 18839) and Queensland (NMW 12329-6 & 12429-8) are based on colonial-era museum vouchers without detailed collection information we consider them unreliable. The records for the Kei islands and New Ireland stem from misidentification of populations of Varanus cf. indicus with high scalecounts, pink tongue and similar dorsal pattern to *Varanus finschi* (Weijola pers. obs.). There is a single record from the Duke of York Islands (AMS R5618) but VW was not able to verify its occurrence there during a field survey in 2012. Thus, as far as we are aware, all verifiable records of *Varanus finschi* are from New Britain.

**Conservation.** The field observations indicate that *Varanus semotus* doesn’t occur, or possibly only at low densities, in the highly degraded secondary forest/bush of large parts of the interior of the island. It is likely that the species occurred throughout Mussau prior to the large scale logging activities of the past three decades (Venter and Arihafa 2015). Thus the species is now mostly restricted to the coastal strip of a relatively small isolated island. Possible threats to the future survival of this species would be the introduction of cane toads which were widely established in the PNG islands during WW2 (Zug et al. 1975). According to unconfirmed accounts by locals they already occur on Emirau Island which also according to local inhabitants on Mussau lack monitor lizards. *Varanus semotus* is the only large-sized terrestrial generalist predator and scavenger on the island, and may well fill an important ecological function, making it of particular conservation concern. The new species is unusual inasmuch as it fills a role normally occupied by Mangrove monitors on isolated Pacific islands and it can well be considered a biogeographical oddity.

## Supplementary Material

XML Treatment for Varanus semotus

## Appendix

**Table:** Scale counts of specimens included in the Principal Components Analysis.

Cataloge nr.	Locality	P	Q	S	T	X	XY	m	N	R
*Varanus doreanus*										
ZMA10193	Sabang	56	107	165	83	57	173	106	88	61
ZMA10194a	Noord R.	55	96	173	82	48	180	110	87	57
ZMA10199	Sermonai R.	43	103	161	92	39	161	95	88	67
ZMA12125	Hollandia	52	100	163	90	44	176	104	95	73
RMNH5164	Digoel R.	54	114	180	97	43	163	120	100	57
RMNH7035	Manokwari	52	102	171	94	40	164	118	97	60
RMNH21029	Gariau-lake	53	113	169	85	42	159	107	87	59
RMNH21051	Fak Fak	49	103	168	89	38	158	118	88	56
RMNH21055b	Manokwari	55	102	158	91	40	153	112	87	56
**Mean**		**52.1**	**104.4**	**167.6**	**89.2**	**43.4**	**165.2**	**110**	**90.8**	**60.7**
*Varanus semotus*										
ZMUT Sa176	Mussau	47	100	161	89	40	153	116	93	74
ZMUT Sa177	Mussau	47	97	162	87	39	147	114	89	66
ZMUT Sa178	Mussau	47	99	152	87	38	149	108	85	67
ZMUC 4272	Mussau	49	103	167	89	39	150	119	92	66
ZMUC 4273	Mussau	51	103	160	89	43	152	118	91	69
**Mean**		**48.2**	**100.4**	**160.4**	**88.2**	**39.8**	**150.2**	**115**	**90**	**68.4**
*Varanus finschi*										
ZMUT Sa186	New Britain	50	106	188	94	50	188	128	92	54
AMR5618	Duke of York	45	103	172	105	54	185	125	98	58
AMR129614	New Britain	49	121	181	99	46	187	131	100	57
MNHN 00 192	New Britain	48	108	174	97	46	165	129	100	45
MNHN 00 195	New Britain	48	108	184	99	51	179	129	98	54
**Mean**		**48**	**109.2**	**179.8**	**98.8**	**49.4**	**180.8**	**128.4**	**97.6**	**53.6**
*Varanus yuwonoi*										
Harvey & Barker (1998)	Halmahera	47	98	174	100	-	-	-	103	
Ziegler et al. (2007a)	Halmahera	53	108	188	101	45	184	137	-	
**Mean**		**50**	**103**	**181**	**100.5**	**45**	**184**	**137**	**103**	
